# COX-2-765G>C Polymorphism Increases the Risk of Cancer: A Meta-Analysis

**DOI:** 10.1371/journal.pone.0073213

**Published:** 2013-09-04

**Authors:** Xiao-feng Wang, Ming-zhu Huang, Xiao-wei Zhang, Rui-xi Hua, Wei-jian Guo

**Affiliations:** 1 Department of Medical Oncology, Fudan University Shanghai Cancer Center, Shanghai, China; Department of Oncology, Shanghai Medical College, Fudan University, Shanghai, China; 2 Department of Medical Oncology, the First Affiliated Hospital of Sun Yat-Sen University, Guangzhou, Guangdong, China; MOE Key Laboratory of Environment and Health, School of Public Health, Tongji Medical College, Huazhong University of Science and Technology, China

## Abstract

**Background:**

Chronic inflammation has been regarded as an important mechanism in carcinogenesis. Inflammation-associated genetic variants have been highly associated with cancer risk. Polymorphisms in the gene cyclooxygenase-2 (COX-2), a pro-inflammation factor, have been suggested to alter the risk of multiple tumors, but the findings of various studies are not consistent.

**Methods:**

A literature search through February 2013 was performed using PubMed, EMBASE, and CNKI databases. We used odds ratios (ORs) with confidence intervals (CIs) of 95% to assess the strength of the association between the COX-2-765G>C polymorphism and cancer risk in a random-effect model. We also assessed heterogeneity and publication bias.

**Results:**

In total, 65 articles with 29,487 cancer cases and 39,212 non-cancer controls were included in this meta-analysis. The pooled OR (95% CIs) in the co-dominant model (GC vs. GG) was 1.11 (1.02–1.22), and in the dominant model ((CC+GC) vs. GG), the pooled OR was 1.12 (1.02–1.23). In the subgroup analysis, stratified by cancer type and race, significant associations were found between the-765 C allele and higher risk for gastric cancer, leukemia, pancreatic cancer, and cancer in the Asian population.

**Conclusion:**

In summary, the COX-2-765 C allele was related to increased cancer susceptibility, especially gastric cancer and cancer in the Asian population.

## Introduction

Cancer is a complicated disease resulting from the combined effect of genetic susceptibility and external elements such as lifestyle and inflammation [1], [2]. The role of inflammation in carcinogenesis is a pivotal issue. Studies have demonstrated that inflammation-associated molecules are associated with a majority of cancer types, and these molecules are activated by various elements related to environment and lifestyle [3]. Signs of inflammation, including cytokines, chemokines, and immune cells, have been identified in many precancerous and cancerous tissues [4]. Several models have typically demonstrated that inflammation induces certain cancers: chronic intestinal inflammation has been associated with colon cancer; *Helicobacter pylori* (HP) with gastric cancer; human papilloma virus (HPV) infection with cervical carcinoma; and hepatitis B virus (HBV) infection with hepatocellular carcinoma [5]–[8]. Chronic inflammation of the colon (e.g., ulcerative colitis) markedly increases the risk of developing colon cancer [9]. The persistent presence of pathogenic microorganisms causes chronic inflammation that raises the likelihood of some cancers [10].

Cyclooxygenase-2 (COX-2), known as prostaglandin-endoperoxide synthase 2 (PTGS2), is a rate-limiting enzyme produced during the production of prostaglandins, and prostaglandins play an important role in inflammation, tumor progression, and metastasis [11]. COX-2 is often undetectable in normal tissue, whereas in tumor tissue specimens its expression is observably higher [12]. It has been reported that COX-2 overexpression contributes to carcinogenesis by increasing cell proliferation, suppressing apoptosis, enhancing invasiveness, and inducing chronic activation of immune responses [13], [14].

Genetic variants may affect the expression of COX-2, and the underlying mechanism is considered to occur through self-regulated transcriptional activity resulting from variations in the capability of its promoter region to bind with certain nuclear proteins [15]. The single-nucleotide polymorphism (SNP) COX-2-765G>C (rs20417) is a functional, extensively studied polymorphism that features guanine (G) converting to cytosine (C) at position-765 bp of the promoter region, altering the transcription activity of the COX-2 gene. Several studies have demonstrated the COX-2-765 G>C polymorphism to be associated with increased risk of human cancers such as gastric cancer, colorectal cancer, prostate cancer, breast cancer, and others [16]–[18]. However, in other studies, the COX-2-765 C allele was not observed to be associated with cancer risk [19]. To further ascertain the relationship between COX-2-765 G>C and cancer risk, several further meta-analyses were performed, but regrettably, the results among studies have varied for different cancer types [20]–[22]. Recently, additional studies of the COX-2-765 G>C polymorphism in several cancer types have been reported; therefore, we conducted this meta-analysis to synthesize the results of these studies and to establish a more durable conclusion.

## Methods

### Publication Search

A systematic literature search through February 12, 2013, was performed using the databases of PubMed and EMBASE and searching for the following terms: (cyclooxygenase-2 or COX-2 or PTGS2) and (polymorphism or polymorphisms or variant or variants or genotype) and (cancer or carcinoma or neoplasm). To expand our investigation, we also searched China National Knowledge Infrastructure (CNKI) database using the following terms in Chinese: COX-2, cancer risk, and polymorphism. References for these articles and eligible literature from review articles were also collected.

### Inclusion and Exclusion Criteria and Data Extraction

Article selection for the meta-analysis used the following inclusion criteria: 1) information on the evaluation of COX-2-765G>C (rs20417) polymorphism and cancer risk; 2) case-controlled study; 3) human subjects; and 4) sufficient genotype data to calculate the odds ratios (ORs) with 95% confidence intervals (CIs). When the same or overlapping populations were included in several publications, the studies with larger sample size were selected. When pertinent data were not included or data presented were unclear, we contacted the authors to collect more data or to clarify the study results. Exclusion criteria were the following: 1) no controls; 2) overlapping study populations; 3) not enough pertinent data; and 4) departure from the Hardy-Weinberg equilibrium (HWE) method in control subjects.

The following data were extracted from all eligible publications: the first author, publication year, cancer type, country and race of the study population, control source (population-based (PB), hospital-based (HB) and family-based (FB)), total number of cases and controls studied, number of cases and controls with the wild-type, heterozygous, and homozygous genotypes, and with the minor allele frequency (MAF). Ethnic subgroups were categorized as Caucasian, Asian, American, and African. For case-control studies with subjects of different races, data were extracted separately for each ethnic group whenever possible. When a study did not include detailed genotypes of each ethnic group, or if it was difficult to discriminate the ethnicity of participants according to the data presented, the study was termed “mixed”. If the study was performed in different counties or regions and the subgroups were indistinguishable in the report, the study was termed “multicenter”. All data were independently extracted by two investigators according to these selection criteria. Disagreement was resolved by discussion.

### Statistical Methods

We utilized odds ratios (ORs) with 95% (confidence intervals) CIs to assess the strength of association between the COX-2-765G>C polymorphism and cancer risk. The pooled ORs with 95% CIs were calculated in a co-dominant model (variant homozygote *vs.* heterozygote) and a dominant model (variant homozygote+heterozygote *vs.* wild-type homozygote). Subgroup analyses were stratified by ethnicity and cancer type.

We used the goodness-of-fit χ^2^ test to evaluate HWE for control subjects in each study, and we considered P<0.05 to representative significant departure from HWE [23]. The heterogeneity assumption was verified using the χ^2^-based Q-test. Q-test results of P>0.05 suggested a lack of heterogeneity among studies, so the pooled OR of all studies was calculated using the fixed-effect model based on the Mantel–Haenszel method. Otherwise, we used the random-effect model, based on the DerSimonian–Laird method, which provides a larger pool of 95% CIs from studies differing among themselves [24], [25].

We also conducted a sensitivity analysis by excluding each study, one at a time, and recalculating the ORs and 95% CIs to assess the effects of each study on the pooled risk of cancer [26]. Then we performed an estimate of potential publication bias using the funnel plot, in which the standard error of log (OR) of every study was plotted against its log (OR) [27], and an asymmetric plot indicated a potential publication bias. We assessed funnel-plot asymmetry using Egger’s linear regression test, a linear regression method of evaluating funnel plot asymmetry on the natural logarithm scale of the OR [28]. The significance of the intercept was determined using the t-test suggested by Egger, and p<0.05 was considered representative of statistically significant publication bias [29], [30]. In cases of publication bias, the Duval and Tweedie nonparametric ‘‘trim and fill.’’ method was performed to adjust for it [31]. All of the statistical tests were performed using STATA version 10.0 (Stata Corporation, College Station, TX).

## Results

### Eligible Studies Characteristics

**Figure 1:**
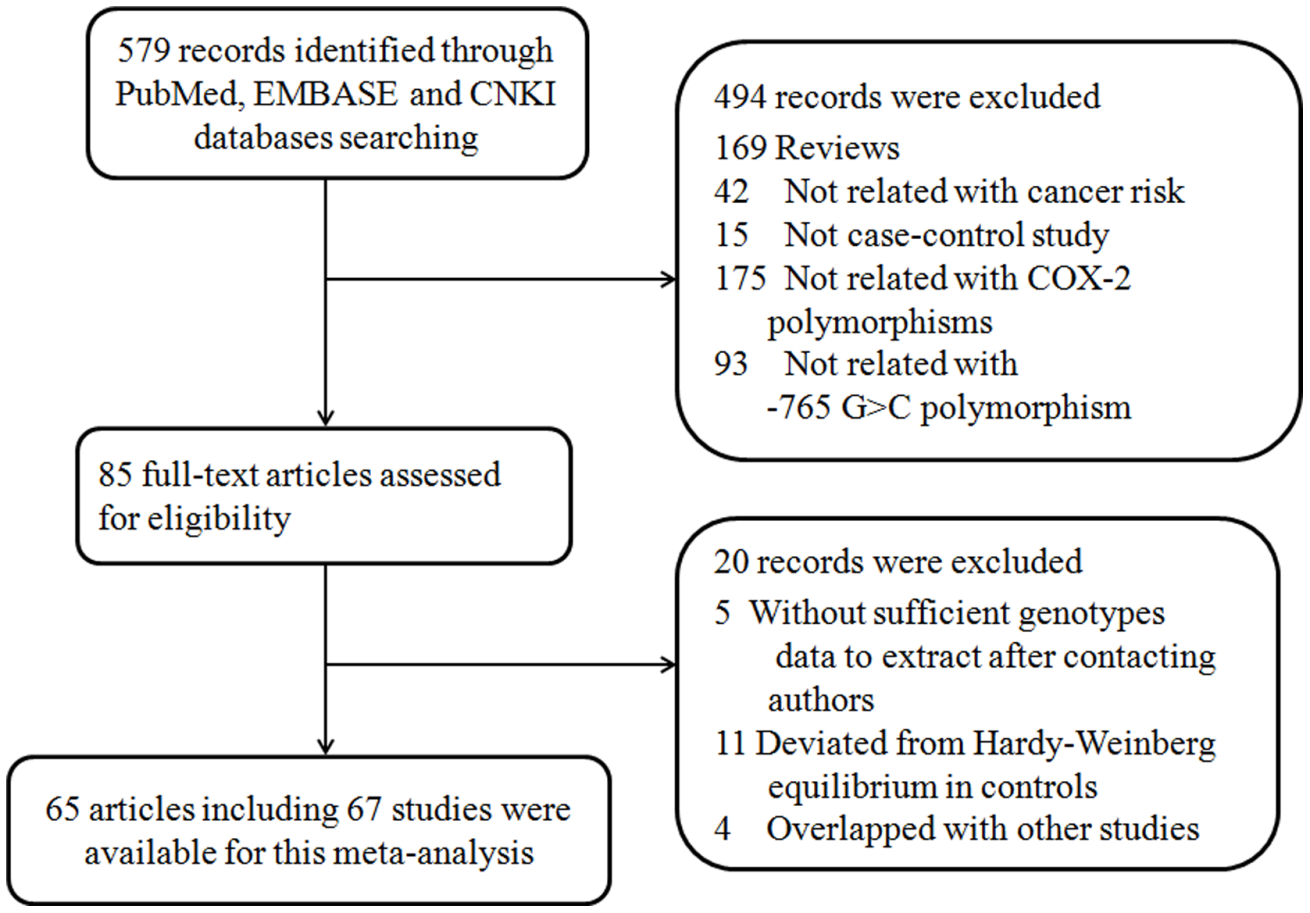
Flow chart of studies selected procedure of this meta-analysis.

A total of 579 publications from the MEDLINE, EMBASE, and CNKI databases were reviewed using the specified key words. After a review of titles and abstracts, 494 publications were excluded according to our criteria. From the remaining 85 studies on COX-2-765G>C polymorphism and susceptibility to cancer that met our inclusion criteria, we eliminated 5 publications due to insufficient genotype data, 11 due to deviation from Hardy-Weinberg equilibrium in controls, and 4 due to overlap with other studies. Finally, 65 articles, including with 29,487 cancer cases and 39,212 non-cancer controls, were included in this meta-analysis. A flow chart of the study selection procedure is shown in **Fig. 1**.

The main characteristics of the studies are listed in **Table 1**. The respective studies focused on the following cancer types: 13 studies investigated colorectal carcinoma [17], [32]–[43], 9 gastric cancer [16], [44]–[51], 8 prostate cancer [18], [52]–[57], 6 esophageal cancer [58]–[62], 3 colorectal adenoma [63]–[65], 4 cancer of head and neck (HNC) [66]–[69], 4 breast cancer [19], [70]–[72], 3 lung cancer [73]–[75], 3 lymphoma [76]–[78], 3 skin cancer [79]–[81], 2 leukemia [82], [83], 2 pancreatic cancer [84], [85], 2 ovarian cancer [86], [87], 2 hepatocellular carcinoma (HCC) [88], [89], 1 cervical cancer [90], 1 glioblastoma [91], and 1 combination of colorectal and esophageal cancers, which were indistinguishable [92]. Twenty-five studies used Asian research subjects, 25 Caucasian, 9 American, 3 African, and 5 used mixed ethnic subjects. Thirty-five study designs were population based (PB), 31 were hospital based (HB), and 1 was family based (FB). The genotyping method used in most of the studies (56/67) was polymerase chain reaction restriction fragment length polymorphism (PCR-RFLP).

**Table 1:** Main characteristics of studies involved in this meta-analysis for an association between COX-2-765 G>C polymorphism and cancer risk.

First author	Year	Cancer type	Country	Race	Study design	Genotype method	Case	Control	MAF^3^ of controls	HWE^4^(P)
Gao	2007	Breast	China	Asian	PB	PCR-RFLP	601	643	0.05	0.70
Cox	2007	Breast	America	mixed	PB	PCR-RFLP	1243	1715	0.17	0.58
Piranda	2010	Breast	Brazilian	American	HB	PCR-RFLP	308	264	0.29	0.21
Dossus	2010	Breast	Multicenter	mixed	PB	PCR-RFLP	6254	8092	0.84	0.13
Tan	2007	Colorectal carcinoma	China	Asian	PB	PCR-RFLP	1000	1300	0.02	0.37
Hamajima	2001	Colorectal carcinoma	Japan	Asian	HB	PCR-CTPP	148	241	0.02	0.70
Xing	2008	Colorectal carcinoma	China	Asian	HB	PCR-RFLP	137	199	0.08	0.84
Koh	2004	Colorectal carcinoma	Singapore	Asian	PB	PCR-RFLP	310	1177	0.05	0.43
Iglesias	2009	Colorectal carcinoma	Spain	Caucasian	HB	PCR-RFLP	284	123	0.21	0.48
Gong	2009	Colorectal carcinoma	America	American	PB	PCR-RFLP	162	211	0.23	0.67
Wang	2012	Colorectal carcinoma	Multicenter	mixed	FB	PCR-RFLP	305	359	0.18	0.49
Daraei	2012	Colorectal carcinoma	Iran	Caucasian	PB	PCR-RFLP	110	120	0.32	0.20
Cox	2004	Colorectal carcinoma	Spain	Caucasian	HB	PCR-RFLP	220	257	0.19	0.73
Hoff	2009	Colorectal carcinoma	Netherlands	Caucasian	HB	PCR-RFLP	326	369	0.17	0.26
Andersen	2009	Colorectal carcinoma	Denmark	Caucasian	PB	QPCR^5^	359	765	0.14	0.61
Pereira	2010	Colorectal carcinoma	Portugal	Caucasian	HB	PCR-RFLP	117	256	0.19	0.37
Thompson	2009	Colorectal carcinoma	America	American	PB	PCR-RFLP	421	479	0.16	0.29
Ulrich	2005	Colorectal adenoma	America	American	PB	PCR-RFLP	494	584	0.17	0.37
Ueda	2008	Colorectal adenoma	Japan	Asian	PB	PCR-RFLP	455	1051	0.03	0.32
Gunter	2006	Colorectal adenoma	America	American	HB	PCR-RFLP	210	196	0.15	0.46
Kristinsson	2009	Esophageal	Netherlands	Caucasian	PB	PCR-RFLP	222	236	0.18	0.47
Upadhyay	2009	Esophageal	India	Asian	HB	PCR-RFLP	174	216	0.18	0.09
Zhang	2005	Esophageal	China	Asian	HB	PCR-RFLP	1026	1270	0.02	0.43
Moons	2007	Esophageal	Netherlands	Caucasian	PB	PCR-RFLP	140	495	0.12	0.24
Bye	2011	Esophageal	South Africa	African	PB	Taqman	347	462	0.51	0.94
Bye	2011	Esophageal	South Africa	mixed	PB	Taqman	190	422	0.32	0.91
Shin	2012	Gastric	Korea	Asian	HB	PCR-RFLP	100	100	0.05	0.60
Li	2012	Gastric	China	Asian	PB	PCR-RFLP	296	319	0.07	0.62
Hou	2007	Gastric	Poland	Caucasian	PB	Taqman	290	409	0.16	0.90
Liu	2006	Gastric	China	Asian	PB	PCR-DHPLC	247	427	0.05	0.27
Tang	2009	Gastric	China	Asian	PB	PCR-RFLP	100	105	0.16	0.11
Zhang	2011	Gastric	China	Asian	PB	PCR-RFLP	357	985	0.02	0.46
Sitarz	2008	Gastric	Netherlands	Caucasian	PB	PCR-sequence	241	100	0.25	0.14
Pereira	2006	Gastric	Portugal	Caucasian	HB	PCR-RFLP	73	210	0.22	0.28
Saxena	2008	Gastric	India	Asian	HB	PCR-RFLP	62	241	0.16	0.42
Chang	2012	HCC^1^	China	Asian	HB	PCR-RFLP	298	298	0.08	0.13
He	2012	HCC	China	Asian	HB	PCR-RFLP	300	300	0.07	0.59
Peters	2009	HNC	Netherlands	Caucasian	HB	PCR-RFLP	428	433	0.14	0.12
Ben	2009	HNC^2^	Tunisia	Caucasian	HB	PCR-RFLP	180	169	0.13	0.93
Mittal	2010	HNC	India	Asian	HB	PCR-RFLP	176	96	0.32	0.08
Chiang	2008	HNC	China	Asian	HB	PCR-RFLP	178	205	0.10	0.13
Wang	2010	Leukimia	China	Asian	HB	PCR-RFLP	266	266	0.06	0.30
Zheng	2011	Leukimia	China	Asian	PB	PCR-RFLP	446	725	0.02	0.56
Coskunpinar	2011	lung	Turkey	Caucasian	HB	PCR-RFLP	231	118	0.50	0.20
liu	2010	lung	China	Asian	HB	QPCR	358	716	0.07	0.06
Campa	2004	lung	Norway	Caucasian	PB	PCR-RFLP	250	214	0.10	0.19
Monroy	2011	Lymphoma	America	American	HB	PCR-RFLP	100	100	0.87	0.48
Hoeft	2008	Lymphoma	Germany	Caucasian	PB	PCR-RFLP	668	661	0.15	0.18
Chang	2009	Lymphoma	America	American	PB	PCR-RFLP	454	354	0.19	0.39
Agachan	2010	Ovarian	Turkey	Caucasian	HB	PCR-RFLP	57	111	0.32	0.38
Pinheiro	2010	Ovarian	Multicenter	mixed	PB	PCR-RFLP	1264	1756	0.17	0.26
Zhao	2009	Pancreatic	China	Asian	PB	PCR-RFLP	393	786	0.02	0.59
Xu	2008	Pancreatic	China	Asian	HB	PCR-RFLP	283	566	0.02	0.61
Cheng	2007	Prostate	America	African	HB	PCR-RFLP	89	88	0.35	0.61
Cheng	2007	Prostate	America	Caucasian	HB	PCR-RFLP	416	417	0.16	0.98
Murad	2009	Prostate	UK	Caucasian	PB	PCR-RFLP	1592	3028	0.16	0.06
Catsburg	2012	Prostate	America	American	PB	PCR-RFLP	1431	756	0.21	0.21
Wu	2011	Prostate	China	Asian	HB	PCR-RFLP	218	436	0.08	0.06
Joshi	2012	Prostate	America	American	PB	PCR-RFLP	935	756	0.21	0.21
Panguluri	2004	Prostate	Nigeria	African	PB	Pyrosequencing	146	108	0.14	0.12
Balistreri	2010	Prostate	Italy	Caucasian	HB	PCR-RFLP	50	125	0.30	0.19
Vogel	2007	Skin	Denmark	Caucasian	PB	QPCR	304	315	0.12	0.93
Lira	2007	Skin	Italy	Caucasian	PB	PCR-RFLP	105	129	0.18	0.59
Cocos	2012	Skin	Romania	Caucasian	HB	PCR-RFLP	174	80	0.22	0.44
Pandey	2010	Cervical	India	Asian	HB	PCR-RFLP	200	200	0.10	0.09
Schwartzbaum	2005	Glioblastoma	Sweden	Caucasian	PB	PCR-DASH	108	399	0.15	0.65
Biramijamal	2011	Colorectal& Esophageal	Iran	Caucasian	PB	PCR-RFLP	60	103	0.18	0.26

1 HCC: hepatocellular carcinoma;

2 HNC: head and neck cancer.

3 MAF: minor allele frequency;

4 HWE: Hardy-Weinberg equilibrium.

5 QPCR: quantitative PCR.

### Quantitive Analysis

**Figure 2:**
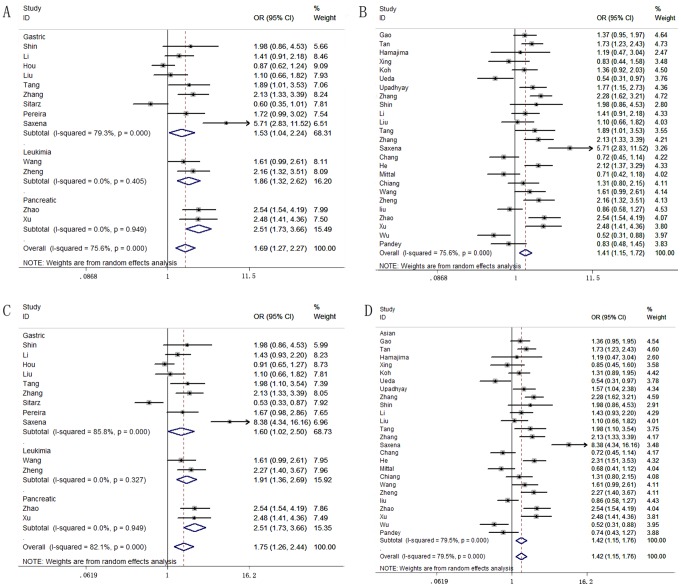
Forest plot of cancer risk associated with the COX-2-765 G>C polymorphism stratified by cancer type and ethnicity. GC vs. GG in the co-dominant model by the random effects for (A) gastric cancer, leukemia, pancreatic cancer and (B) in the Asian population. (CC+GC) vs. GG in a dominant model by the random-effects for (C) gastric cancer, leukemia, pancreatic cancer and (D) in the Asian population.

The main results of the meta-analysis are listed in **Table 2**. The association between COX-2-765 G>C polymorphism and cancer risk was estimated in two comparison models: a co-dominant model (GC vs. GG) and a dominant model ((CC+GC) vs. GG). The analysis used a random pooling model because the heterogeneity among studies was significant in the co-dominant model and in the dominant model (p<0.001). In the co-dominant model, the overall pooled effect indicated that the-765 GC heterozygote was associated with a significantly increased overall cancer risk, compared with the GG homozygote (OR = 1.11, 95% CI = 1.02–1.22, P = 0.01). In stratification analyses by cancer type and ethnicity, the association was maintained in gastric cancer (OR = 1.53, 95% CI = 1.04–2.24, p = 0.03), leukemia (OR = 1.86, 95% CI = 1.32–2.62, P<0.01), pancreatic cancer (OR = 2.51, 95% CI = 1.73–3.66, P<0.01), and cancer in the Asian population (OR = 1.41, 95% CI = 1.16–1.72, p<0.01) (**Fig. 2A and B**). Notably, the association between the COX-2-765 C allele and decreased cancer risk was found in the Caucasian population (OR = 0.91, 95% CI = 0.83–1.00, P = 0.04). However, this difference may have been the result of different ethnic subjects and bias from different genotyping methods. In the dominant model, we found significant associations of this SNP with cancer risk in overall cancer susceptibility (OR = 1.12, 95% CI = 1.02–1.23, P = 0.01), gastric cancer (OR = 1.60, 95% CI = 1.02–2.50, P = 0.04), leukemia (OR = 1.91, 95% CI = 1.36–2.69, P<0.01), pancreatic cancer (OR = 2.51, 95% CI = 1.73–3.66, P<0.01), and cancer in the Asian population (OR = 1.42, 95% CI = 1.15–1.76, P<0.01) (**Fig. 2C and D**).

**Table 2:** Quantitive synthesis of the associations between COX-2-765 G>C polymorphism and cancer risk in two models.

	No of studies	GC vs. GG OR (95%CI)	P	P_heterogeneity_	CC+GC vs. GG OR (95%CI)	P	P_heterogeneity_
**Total**	67	**1.11(1.02–1.22)**	**0.01**	**0.00**	**1.12(1.02–1.23)**	**0.01**	**0.00**
**Cancer type**							
Gastric	9	**1.53(1.04–2.24)**	**0.03**	**0.00**	**1.60(1.02–2.50)**	**0.04**	**0.00**
Leukemia	2	**1.86(1.32–2.62)**	**0.00**	**0.41**	**1.91(1.36–2.69)**	**0.00**	**0.33**
Pancreatic	2	**2.51(1.73–3.66)**	**0.00**	**0.95**	**2.51(1.73–3.66)**	**0.00**	**0.95**
Colorectal carcinoma	13	1.03(0.88–1.21)	0.58	0.02	1.04(0.90–1.20)	0.60	0.06
Prostate	8	0.92(0.77–1.10)	0.36	0.01	0.90(0.75–1.70)	0.22	0.01
Esophageal	6	1.28(0.89–1.84)	0.19	0.00	1.31(0.92–1.87)	0.14	0.00
HNC	4	1.11(0.80–1.54)	0.52	0.09	1.10(0.76–1.60)	0.60	0.03
Breast	4	0.95(0.80–1.132)	0.58	0.12	0.97(0.82–1.14)	0.70	0.12
Colorectal adenoma	3	0.88(0.63–1.23)	0.45	0.13	0.87(0.64–1.20)	0.84	0.16
Skin	3	1.01(0.74–1.22)	0.94	0.30	1.06(0.81–1.39)	0.69	0.36
Lung	3	0.86(0.66–1.12)	0.27	0.43	0.80(0.57–1.13)	0.20	0.57
Lymphoma	3	0.98(0.75–1.27)	0.86	0.23	0.99(0.71–1.40)	0.96	0.11
HCC	2	1.24(0.43–3.58)	0.70	0.00	1.29(0.41–4.07)	0.66	0.00
Ovarian	2	1.62(0.48–5.51)	0.44	0.00	1.47(0.52–4.17)	0.47	0.00
Other*	3	1.30(0.61–2.76)	0.49	0.01	1.33(0.53–3.32)	0.55	0.00
**Ethnicity**							
Asian	25	**1.41(1.16–1.72)**	**0.00**	**0.00**	**1.42(1.15–1.76)**	**0.00**	**0.00**
Caucasian	25	1.04(0.92–1.17)	0.53	0.00	1.04(0.92–1.18)	0.51	0.00
African	3	0.67(0.35–1.27)	0.22	0.02	0.64(0.35–1.18)	0.15	0.02
American	9	1.03(0.93–1.13)	0.58	0.65	1.02(0.93–1.12)	0.69	0.55
Mixed	5	**0.91(0.83–1.00)**	**0.04**	**0.84**	0.93(0.85–1.02)	0.10	0.51

* Cancers studied in only one article were combined and termed “other.”

### Heterogeneity, Sensitivity Analysis, and Publication Bias

Heterogeneity was determined using the χ^2^-based Q-test, and heterogeneity was found in two pooling models (P<0.01 in both models), so the random model was utilized to generate a larger pool of studies with 95% CIs. We performed the sensitivity analysis by assessing the influence of an individual study on the overall OR. No individual study affected the pooled OR markedly, since omission of any single study made no substantial difference. Also, we conducted Begger’s funnel plot and Egger’s test to assess the publication bias of all eligible literature. The shapes of the funnel plot seemed symmetrical in two comparison models, and statistical results from Egger’s test still did not show publication bias (p = 0.36 in co-dominant model and p = 0.34 in dominant model). These findings demonstrated that publication bias, if any, did not significantly affect the results of our meta-analysis for the association between COX-2-765G>C and cancer risk.

## Discussion

COX-2-765G>C is a functional polymorphism, located at 765 bp upstream (-765 bp) from the transcription start site. It changes a putative stimulatory protein (Sp1) binding site in the promoter of COX-2 between-766 and-761 bp [93], but it creates an E2 promoter factor (E2F) binding site, leading to high transcription activity, which may be the mechanism of COX-2-765G>C polymorphism increasing cancer risk [15].

The current meta-analysis explored the role of COX-2-765G>C polymorphism in the susceptibility of cancer among 65 articles with 29487 cancer cases and 39212 non-cancer controls. We found that C-allele carriers had an increased risk of cancer, especially gastric cancer, leukemia, and pancreatic cancer and cancer in the Asia population, when compared with G carriers. Our results show that COX-2-765 C carriers are at significantly increased risk for gastric cancer, leukemia, and pancreatic cancer but not other cancer types. One possible explanation is that different types of cancer have various mechanism of carcinogenesis. Additionally, it is possible that the significant difference effects are casual, because studies with small sample sizes have deficient statistical power to disclose a slight effect. Interestingly, our meta-analysis revealed an association between the COX-2-765 C allele and decreased cancer risk in Caucasian population. In this Caucasian subgroup, a large study sample with 6254 cases and 8092 controls (two thirds of all subjects in this subgroup) showed an MAF (0.84) [72] significantly higher than in other reports, which may have affected the results. Additionally, this extremely high MAF value may have resulted from bias induced by experimental procedure and methods. Our study differed from previous meta-analyses in the subgroup analysis of gastric and colorectal cancer. Zhu reported a significant association between-765G>C polymorphisms and colorectal carcinoma, but not in gastric cancer, contrary to the results of our present study [94]. In other studies, researchers analyzed the role of COX-2-765G>C polymorphism in diverse cancer types. No convincing association between the C allele and risk of prostate cancer [22], [95], breast cancer [21], and colorectal cancer [96] respectively, were revealed, but a significant association was reported between C allele and risks for gastric cancer [97] and esophageal cancer [98]. However, the number of subjects included in previous studies was not as large, and our meta-analysis includes the latest studies. Furthermore, we analyzed at least twice as many studies as meta-analyses published previously [94]. In summary, our findings provide the most current and powerful conclusion among analyses of this type.

Limitations encountered in this analysis should be considered as these results are interpreted. First, the CC genotype frequency in many studies was zero, so we assumed a co-dominant model and a dominant model. For some polymorphisms, this model might not be the most suitable for a clear assessment of the gene–disease interaction. Secondly, the results of the subgroup stratification analysis must be interpreted with caution because of the limited number of published studies. For example, only two reports for leukemia and pancreatic cancer were included. Thirdly, there is marked heterogeneity among studies in overall and some subgroup analyses, which may derive from ethnic groups and types of cancer, may have skewed our results. Finally, this systematic review was based on unadjusted data, as the genotype information stratified for the main confounding variables was not available in the original papers and the confounding factors addressed across the different studies varied. Adjusted estimates might provide more precise and stronger associations, as they reduced the impact of possible confounding factors. To determine a precise association between the COX-2-765G>C and cancer genetic susceptibility, it is essential to design and perform scientific and rigorous studies with large sample sizes in the future.

Although further research is needed, this present meta-analysis validates a significant association between COX-2-765G>C polymorphism and genetic cancer susceptibility, especially in gastric cancer, leukemia, pancreatic cancer, and cancer in the Asian population. If confirmed in future studies, this genotype may be used by clinicians to select individuals for early diagnosis and treatments.
